# Supplementary material for: Health-Nutrients and Origin Awareness: Implications for Regional Wine Market-Segmentation Strategies Using a Latent Analysis
Source: Nutrients. 2022 Mar 26;14(7):1385. doi: 10.3390/nu14071385 (PMC9002975; doi:10.3390/nu14071385)
Supplement: Supplementary file 1 [file nutrients-14-01385-s001.zip › nutrients-1642425-supplementary.pdf]

Supplementary File S1

Table S1: Consumers' classes profile

Characteristic	Class 1<br>n = 185<br>(42 %)	Class 2<br>n = 65<br>(15 %)	Class 3<br>n = 78<br>(18 %)	Class 4<br>n = 11<br>(25%)
**Behavior & propensity to wine consumption & purchase**				
	%	%	%	%
**Consumption frequency**				
More than once a week	66.48	58.46	67.94	62.16
Less than one a week	33.52	41.54	32.06	37.84
**Consumption metrics**				
More than half-liter each day	38.91	26.15	41.02	36.03
Less than half-liter each day	61.09	73.85	58.95	63.97
	Mean	Mean	Mean	Mean
Number of glasses per week (usually out of meals)	1.04	1.00	1.74	1.24
**Consumption place (as usual drinker)**	%	%	%	%
At proper, parents and/or friends' house(s)	50.27	29.23	41.02	49.54
At restaurants, pubs, wine bars and/or night-clubs	49.73	70.77	59.98	50.46
**Purchase place**				
Specialists wine shops	35.15	43.07	38.46	46.84
Hypermarkets, supermarkets, large non specialized stores, hard discount, via internet	64.87	56.93	61.54	53.16
**Purchase choice parameter importance***	Mean	Mean	Mean	Mean
Quality	2.62	2.67	2.57	2.68
Price	2.22	2.30	2.30	2.20
Alcohol content	1.85	1.75	1.93	2.01

Characteristic	Class 1<br>n = 185<br>(42.14 %)	Class 2<br>n = 65<br>(14.80 %)	Class 3<br>n = 78<br>(17.76 %)	Class 4<br>n = 11<br>(25.28 %)
**Behavior & propensity to wine consumption & purchase (Cont.)**				
**Purchase choice parameter importance* (Cont.)**	Mean	Mean	Mean	Mean
Label aspects	1.77	1.90	1.83	1.99
Label content	2.17	2.33	2.12	2.35
Origin of wine grapes	2.56	2.46	2.52	2.50
Cultivar	2.41	2.10	2.21	2.36
**Simulation on the wine purchase**				
**Health claims importance* on the label**	Mean	Mean	Mean	Mean
The grapes come from Apulian autochthonous vines	2.66	2.53	2.53	2.31
Wine is produced with typical local Apulian vines	2.37	2.26	2.41	2.15
Wine is produced with ancient Apulian vines	2.35	2.27	2.43	2.23
Wine is produced with natural autochthonous yeasts	1.96	2.00	2.14	1.81
Wine is produced with low content of sulphites	2.45	2.60	2.42	2.35
Wine with low alcohol degree	1.43	1.61	1.71	1.62
Wine is produced with high content of antioxidants	2.07	2.35	2.21	1.95
Wine is produced through integrated production system (low ecological impact)	2.38	2.53	2.35	2.27

Characteristic	Class 1<br>n = 185<br>(42.14 %)	Class 2<br>n = 65<br>(14.80 %)	Class 3<br>n = 78<br>(17.76 %)	Class 4<br>n = 11<br>(25.28 %)
**Socio-economic characteristics & Health status**				
	%	%	%	%
**Sexe**				
Male	41.08	50.76	47.43	54.95
female	58.92	49.24	52.57	45.05
	Mean	Mean	Mean	Mean
**Age**	45.47	41.67	41.00	47.00
**Civil status**	%	%	%	%
Married or cohabitant	17.83	40.00	34.61	24.32
Single, divorced or separate	82.17	60.00	65.39	75.68
**Level of education**	Mean	Mean	Mean	Mean
**(in year)**	16.98	17.18	15.85	15.55
**Annual income**	Mean	Mean	Mean	Mean
**(in thousand Euro/year)**				
Less than 20	20.00	26.15	37.17	22.52
Between 20.1 and 35	48.10	40.00	44.87	53.15
More than 35	31.89	33.84	17.94	24.32
	Mean	Mean	Mean	Mean
**Body mass index**	24.24	23.87	23.93	24.48
**Disease affectation**	%	%	%	%
Disease of the cardiovascular system	4.86	1.53	2.56	4.50
Disease of the respiratory system				
Disease of the immune system	3.78	4.61	3.84	2.70
Disease of the nervous system	3.24	3.07	5.12	1.80
Metabolism disease	3.24	0.00	0.00	0.00
Other diseases	8.10	0.00	1.28	6.30
	9.72	9.23	8.97	10.81
**General health status level****	Mean	Mean	Mean	Mean
	1.08	1.29	1.52	1.02

*Importance scoring of choice parameter: 1: high importance; 2: medium importance; 3: least importance

** Health status level: 1: Very good; 2: Good; 3: Normal; 4: Bad; 5: Very bad
